# Pressure-driven supercritical CO_2_ transport through a silica nanochannel†

**DOI:** 10.1039/c7ra11746a

**Published:** 2018-01-04

**Authors:** Bing Liu, Xiaoqi Li, Chao Qi, Tingyi Mai, Kaiyun Zhan, Li Zhao, Yue Shen

**Affiliations:** School of Science, China University of Petroleum Qingdao 266580 Shandong China liubing19720115@gmail.com zhanky@upc.edu.cn

## Abstract

A thorough understanding of supercritical CO_2_ (scCO_2_) transport through nanochannels is of prime significance for the effective exploitation of shale resources and the mitigation of greenhouse gas emission. In this work, we employed the non-equilibrium molecular dynamics simulations method to investigate the pressure-driven scCO_2_ transport behavior through silica nanochannels with different external forces and pore sizes. The simulations reveal that the capability of scCO_2_ diffusion enhances both in the bulk region and the surface adsorbed layer with the increasing of pressure gradient or nanochannel size, in addition, the slip length increases nonlinearly with the external acceleration or nanochannel width increases and finally reaches a maximum value. The negative slippage occurs at lower pressure gradient or within the narrower nanochannel. Overall, it is the combined effect of strong adsorption, surface diffusion and slippage that causes the nonlinear relation between flow rate and pressure gradient or nanochannel size. The present work would provide theoretical guidance for the scCO_2_ enhanced shale oil/gas recovery, CO_2_ storage, and mass transport in nanoporous materials.

## Abstract 2

The mechanism of pressure-driven scCO_2_ transport through silica nanochannels with different external force and pore size was invested.

## Graphical abstract

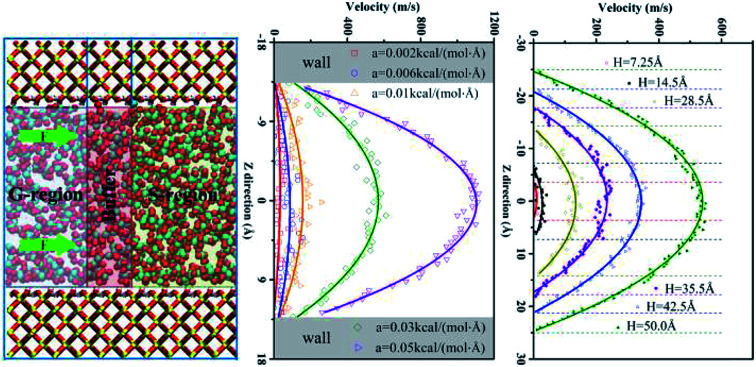

## 1. Introduction

Recently, the behaviors of supercritical CO_2_ (scCO_2_) in narrow pores and confined spaces have attracted extensive attention in the exploration and development of hydrocarbons stored in shale,^1,2^ such as scCO_2_ fracturing^3,4^ and injecting.^5,6^ On the other hand, CO_2_ injection into tight shales is one of the promising solutions for mitigating carbon emissions^7,8^ and global warming.^9,10^ The nanopores are widely present in shale reservoirs.^11–14^ The basis for scCO_2_ geosequestration^15,16^ and enhanced hydrocarbon recovery depends on its unique transport properties induced by the confinement of shale nanopores. The evaluation of scCO_2_ enhancing hydrocarbon recovery, enhancement of fracturing efficiency, and estimation of storage capacity require understanding of microscopic behaviors of scCO_2_ through shale nanopores, such as adsorption, diffusion and flow.

Confined fluids in nanopores demonstrate microstructural, dynamical and thermophysical behavior that differ significantly from their bulk counterparts due to the molecular asymmetry between fluid–fluid and solid–fluid interactions.^17^ This asymmetry leads to inhomogeneous local fluid density distributions, where the superimposition of the attractive potentials from two walls induces confined environments.^18^ Extensive molecular simulation studies were reported on CO_2_ sorption in the graphite slit-pores^18–24^ usually treated as the organic matrix of shale reservoirs. In addition, several molecular simulations were addressed on the adsorption structure of CO_2_ confined in clay-like^25,26^ and silica^17,27^ slit pores. The results revealed that the nanopore size dramatically influences the adsorption structure of CO_2_, which would has potentially significant effects on the transport properties of CO_2_ inside the nanopore.^28^ The diffusion of confined CO_2_ obviously weakens as compared with that in bulk fluid^25,27^ and CO_2_ diffusion coefficient reduces with the decrease in the pore size.^25^ The diffusion and migration of CO_2_ in silica nanopore occur predominantly along the surface due to hydrogen bonds formed between CO_2_ and the surface –OH group.^27,29^ Besides, the widely developed nanopores and ultra-low permeability in shale reservoirs raise concerns on the applicability of Darcy's law to account for mass transport in shale. However, it breaks down for flow through very narrow pores because the continuum flow hypothesis is invalid.^23,28^ Efforts have been made to moderate for the breakdown of Darcy's law *via* gas slippage theory,^23^ for example, the Klinkenberg effect.^30,31^ Such empirical corrections can not capture the complex adsorption and transport behavior of the fluid in ultra-confining porous materials.^32^ Falk *et al.* demonstrated that the non-Darcy behavior results from strong adsorption in kerogen and the breakdown of hydrodynamics at the nanoscale.^32^ Monteiro *et al.* suggested a hydrodynamic model of gas flow in nanoporous media by introducing a pressure gradient-dependent permeability of kerogen, which obeys power function relationship.^33^ Wang *et al.* reported the pressure-driven flow behavior of scCO_2_ confined in shale organic nanopores.^24^ They observed that the streaming velocity profile tends to a plug flow and the magnitude far exceeds the prediction of the no-slip Poiseuille equation. Firouzi and Wilcox investigated the gas slippage and Klinkenberg effects of CO_2_ confined in carbon slit pores.^23^ They reported a plug flow occurs at pore size less than 2 nm, a parabolic velocity profile at pore size larger than 10 nm, and the dramatic underestimation of the CO_2_ permeability using the bulk phase viscosity. In summary, the transport of scCO_2_ in inorganic nanochannel remains unclear, and there is a strong need for a reliable theoretical framework of scCO_2_ transport inside nanoporous matrix due to the constraint of the lowest permeability in the fluid path to the overall permeability of the shale.

Herein, non-equilibrium molecular dynamics (NEMD) simulations were conducted to investigate the pressure-driven scCO_2_ transport through the silica nanochannel. The effects of flow driving pressure gradient and nanochannel size on scCO_2_ transport were determined by exploring the adsorption, diffusion and flow of scCO_2_ molecules. The density profiles and residence autocorrelation functions were employed to describe adsorption. The velocity profiles, the slippage effect, and the flow rate as the function of pressure gradients or nanochannel sizes were applied to characterize the flow behavior.

## 2. Models and methodology

**Fig. 1:**
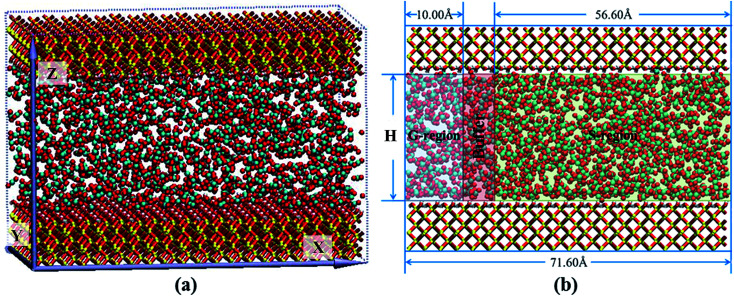
The MD simulation model: (a) snapshot of simulation model. (b) Schematic images of different region distribution.

The nanochannel with the size of 71.60 × 50.12 × *H* Å^3^ was formed by two 10.5 Å cristobalite plates. *H* denoted the interlayer space of two plates providing the nanochannel for scCO_2_ transport. The silica nanochannels with hydroxylated surfaces^34^ were fixed in all simulations. Periodic boundary conditions were applied in three dimensions. To obtain saturated adsorption of scCO_2_ in nanochannels under 373.15 K and 35 MPa, equilibrium molecular dynamics (EMD) simulations were carried out for 2 ns to get the isothermal adsorption curve^35^ (ESI Fig. S1†). The initial model was shown in Fig. 1(a).

As applying an acceleration or a force on all atoms directly^24,36–38^ was improper to simulate desorption from surfaces, the whole nanochannel was divided into three segments along *X* dimension, named G-region, Buffer and S-region, respectively, as shown in Fig. 1(b). A driving pressure gradient was exerted on the system by applying an acceleration along *X* direction on scCO_2_ molecules in G-region in order to generate the Poiseuille flow through the nanochannel. A bigger acceleration corresponds to a greater pressure gradient. The effect of pressure gradient on transport behavior was examined by altering the acceleration ranged from 0.001 to 0.05 kcal (Å^−1^ g^−1^) as *H* is 28.50 Å. The effect of nanochannel sizes on transport behavior was studied by altering *H* from 7.25 to 50.00 Å as the acceleration is 0.01 kcal (Å^−1^ g^−1^). All calculations were executed in S-region. The NEMD simulations were performed to simulate mass transport for 6 ns which is enough to obtain a steady scCO_2_ flow.

Both the EMD and NEMD simulations were performed using large-scale atomic/molecular massively parallel simulator (LAMMPS)^39^ software in the NVT ensemble with a time step of 1 fs. Visualization of the dynamics process was carried out using the program VMD.^40^ The Nosé–Hoover^41,42^ thermostat was employed to control the temperature. The silica nanochannel was modeled with the CLAYFF^43^ force field. CO_2_ molecules were described by the three-site EPM2 (ref. 44) model which was successfully demonstrated to reproduce critical point of CO_2_ in experiment. All the VDW interactions were described by the 12–6 Lennard-Jones potential. The cutoff of non-bonded interaction was set to be 9.5 Å. Long-range electrostatic interaction was calculated by PPPM^45^ summation method with the accuracy of 0.0001e. The interaction between different types of atoms was determined by the Lorentz–Berthelot rule:
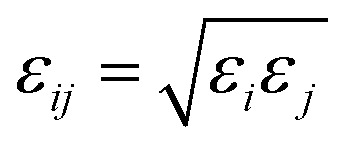
 and *σ*_*ij*_ = (*σ*_*i*_ + *σ*_*j*_)/2. All parameters were given in the ESI Table S1.†

## 3. Results and discussions

### 3.1 Effect of pressure gradient

#### 3.1.1 Effect of pressure gradient on adsorption of scCO_2_

**Fig. 2:**
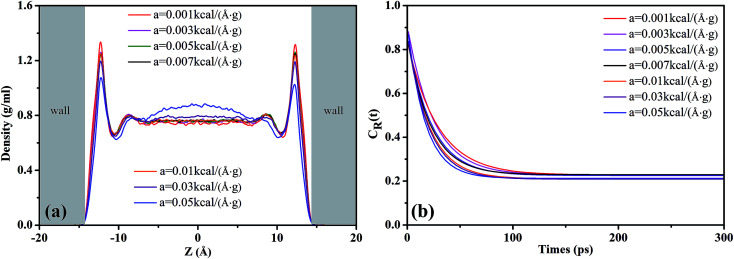
Adsorption of scCO_2_ (a) density profiles of scCO_2_ in 28.50 Å SiO_2_ nanochannel with external acceleration ranging from 0.001 kcal (Å^−1^ g^−1^) to 0.05 kcal (Å^−1^ g^−1^). (b) The residence autocorrelation function for carbon atoms in the adsorbed layers as a function of time under different acceleration.

In Fig. 2(a), we presented the density profiles of scCO_2_ under different pressure gradients to explore the influence of pressure gradient on adsorption. As shown, the density profiles along *Z* direction are symmetrical about the central axis of the nanochannel. There are two primary peaks and two secondary peaks appear near the surfaces, indicating that the scCO_2_ form four structuring adsorption layers. This shows that the scCO_2_ molecules pack more closely near the surface than those far away from the surfaces. The monolayer peak positions are almost independent of pressure gradient, while the second layer peaks shift slightly as the gradient pressure increases and almost disappears at the highest gradient pressure. The density in the center of the channel increases slightly under relatively low pressure gradients while increases obviously at higher pressure gradients. The results indicate that a high pressure gradient dramatically affects the distribution of scCO_2_ inside the nanochannel. Under the high pressure gradients, some of adsorbed scCO_2_ desorb from the surfaces and enter into the channel central areas. Such behavior could improve the effective permeability^46^ of scCO_2_ in channel, which contributes to the scCO_2_ flow. However, the shape of the density profiles remains almost unchanged under the lower pressure gradients indicating a little influence on the distribution of scCO_2_, which is in agreement with the results obtained by Kasiteropoulou.^47^

In order to quantify the residence time of scCO_2_ molecules remaining near the nanochannel surface, residence autocorrelation function, *C*_R_(*t*), was calculated by:^48–50^1
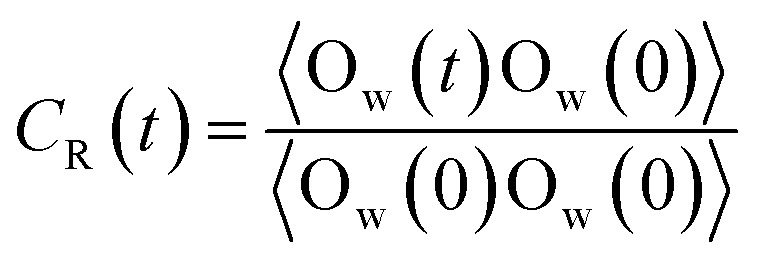
where O_w_(*t*) describes whether one scCO_2_ molecule is in the first adsorption layer at time *t*. O_w_(*t*) equals one indicating a scCO_2_ molecule within the first adsorption layer. O_w_(*t*) equals zero meaning that a scCO_2_ molecule leaves the first adsorption layer. The angular bracket denotes the ensemble average. Carbon atom in scCO_2_ is considered to identify the position of one scCO_2_ molecule. *C*_R_(*t*) decays from 1 to 0. The faster decay of *C*_R_(*t*) implies the shorter time for scCO_2_ residing in the first adsorption layer.

The *C*_R_(*t*) for scCO_2_ at the different pressure gradients is shown in Fig. 2(b). *C*_R_(*t*) decreases faster as pressure gradient increases, implying a shorter residence time of scCO_2_ in the adsorption layer at bigger pressure gradients. The high pressure gradients could facilitate the fluid mobility and enhance the occurrence of molecular collisions, and then driving the adsorbed scCO_2_ molecules away from the surface. The *C*_R_(*t*) plateaus occurs after about 120 ps, indicating that some of the adsorbed scCO_2_ molecules are not desorbed within the simulation time because of the electrostatic interaction and the hydrogen bond between scCO_2_ and the surface.^51^ These scCO_2_ molecules that can not be desorbed from the surfaces will reduce the scCO_2_ flow in shale reservoirs but benefit the CO_2_ sequestration.

#### 3.1.2 Effect of pressure gradient on diffusion coefficient of scCO_2_

The diffusion coefficient is a significant parameter revealing the mechanism of mass transfer. The self-diffusion coefficient can be obtained by Einstein's relation2
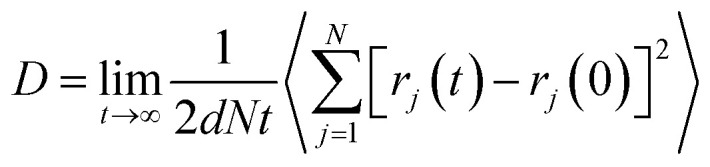
where *r*_*j*_ is the position vector of the *j* scCO_2_ molecule, *d* is the dimension of the system, *N* is the total scCO_2_ molecules and the angular bracket represents the ensemble average. In this work, *D* was calculated for motions in *Y* and *Z* dimensions, and hence *d* = 2.

**Fig. 3:**
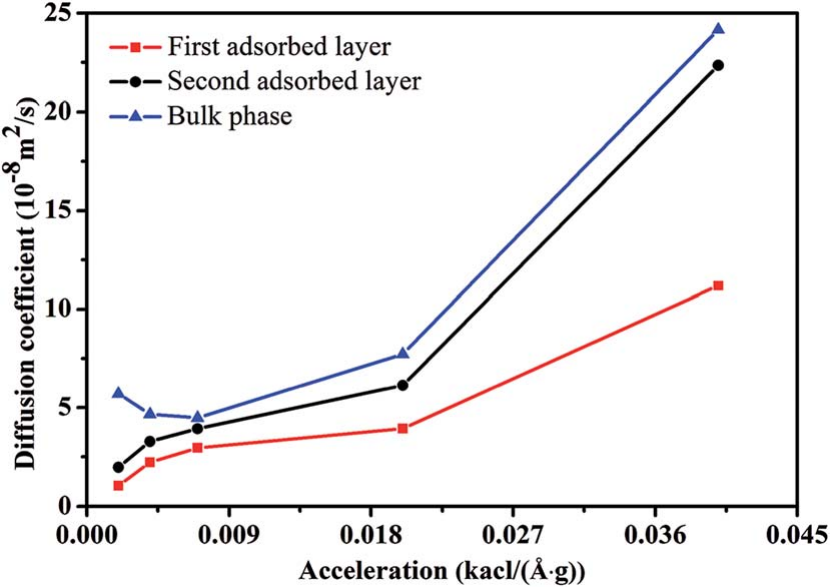
The diffusion coefficient of scCO_2_ molecular in different adsorbed layers with different external accelerations.

Fig. 3 illustrates the diffusion coefficients of scCO_2_ at different pressure gradients and areas in the nanochannel. As pressure gradient increases, the diffusion coefficient of scCO_2_ in the adsorption layers increases while it decreases firstly and then rises for bulk scCO_2_. The variation of diffusion coefficients of scCO_2_ under lower pressure gradients was mainly affected by the variation in the density distribution. As shown in Fig. 2(a), the density near the surface reduces with the increasing pressure gradient as the acceleration is less than 0.007 kcal (Å^−1^ g^−1^). This indicates that the partial pressure in adsorbed layers decreases, leading to the increase of scCO_2_ diffusion coefficient. The increasing density of bulk scCO_2_ indicates the enhancement of the partial pressure in the central area of nanochannel, resulting in the decrease of scCO_2_ diffusion coefficient. In this case, the density distribution plays the dominant role in the diffusibility of scCO_2_ within the nanochannel. As the acceleration is greater than 0.007 kcal (Å^−1^ g^−1^), the largely undermined adsorption strength with increasing pressure gradient indicates the increase of the scCO_2_ velocity within the nanochannel. The raising velocity may lead to the decrease of scCO_2_ pressure, which induces the increase of the diffusion coefficient. Herein, the increasing velocity plays the pivotal role in scCO_2_ diffusibility inside the nanochannel. As indicated from the Stokes–Einstein relation, the increase in the diffusion coefficient implies the decrease in the viscosity. Therefore, we conclude that such changes could affect the flow of scCO_2_ within the nanochannel.

#### 3.1.3 Effect of pressure gradient on flow behavior of scCO_2_

**Fig. 4:**
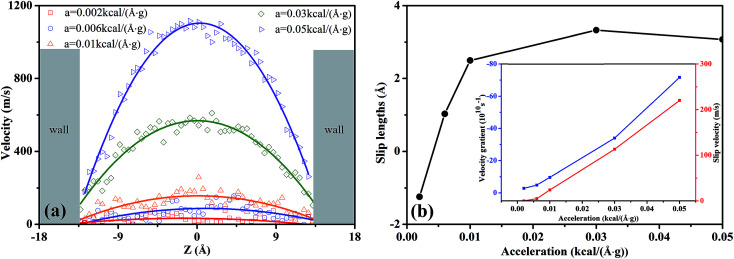
(a) The effects of external acceleration on the velocity profiles of scCO_2_ molecular in nanochannel. (b) Slip length as a function of the external acceleration for scCO_2_ molecular.

In Fig. 4(a), we depicted the velocity profiles at different pressure gradients in order to characterize the flow behavior of scCO_2_ within the nanochannel. The velocity profiles were obtained by fitting data using the quadratic function:

3 *v*(*z*) = *az*^2^ + *bz* + *c*

where *a*, *b*, and *c* are the fitting parameters describing the flow process, *a* characterizes the curvature. The lager the absolute value of *a* is, the steeper the curve and the greater the velocity gradient. *c* represents the maximum velocity. The fitting parameters were provided in the ESI Table S4.†

In Fig. 4(a), the velocity profiles with the parabolic shape^24,36,38^ are symmetrical about the central axis of the nanochannel. The larger pressure gradients induce the greater axial velocities and sharper parabolic velocity curves. Moreover, we notice that there are non-zero velocities near the surfaces, which leads to the slippage phenomenon. The slip velocity increases as the pressure gradient increases. Here, we employed the slip length *L*_s_ to describe the slippage phenomenon, as defined by^52^4
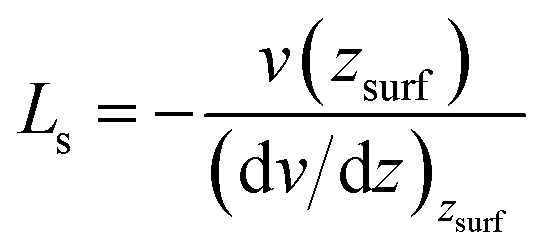
where *v*(*z*_surf_) is the slip velocity, *v* is the velocity profile in the flow direction, and (d*v*/d*z*)_*z*_surf__ is the velocity gradient with respect to *z* on the surface. Slip length is the distance extrapolated into the surface where the velocity vanishes as supposed by no-slip boundary condition.^53^Fig. 4(b) demonstrates the slip lengths for scCO_2_ flow in silica nanochannel under different pressure gradients. The inset shows the derivatives of the velocity with respect to *z* on the surface and the slip velocities. Clearly, a negative slip length^54–57^ occurs as the acceleration is less than 0.004 kcal (Å^−1^ g^−1^). The main reason can be explained as follows. Under small pressure gradients, the shear force, as reflected by velocity gradient in the inset in Fig. 4(b), is too small to overcome the attractive interactions between scCO_2_ and the surface. This leads to the zero velocity for scCO_2_ molecules near the surfaces, indicating the presence of scCO_2_ boundary layer whose thickness equals slip length. In this case, adsorbed scCO_2_ are trapped on nanochannel surfaces, which is so-called sticking phenomenon,^58^ confining the flow and diffusion of scCO_2_. In addition, the slip length shows a non-monotonous increase with pressure gradients. The increasing pressure gradient promotes scCO_2_ flow and detaches scCO_2_ molecules from the surfaces due to the enhancement of shear rate, which stimulates surface diffusion^59^ of scCO_2_. Moreover, the rate of increase in slip velocity is greater than that of velocity gradient. Consequently, slip length increases rapidly with increasing pressure gradient. As pressure gradient further increases, the ratio of slip velocity to velocity gradient decreases gradually which cause the slip length increases slowly and reaches a maximum value finally. After that, the velocity of bulk scCO_2_ increases much faster than slip velocity with the increase in pressure gradient due to the strong adsorbed potential of the surfaces. This leads to a large increase in velocity gradient greater than the rate of increase in slip velocity. Therefore, slip length will decrease gradually as pressure gradient increases.

**Fig. 5:**
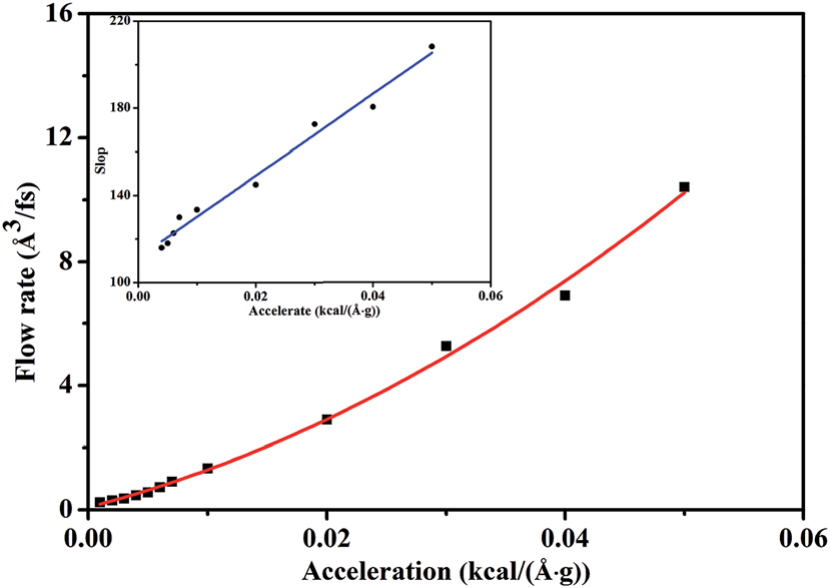
The flow rate as a function of the external acceleration for scCO_2_ molecular.

Fig. 5 depicts the scCO_2_ flow rate as a function of pressure gradient, revealing a nonlinear relationship different from Darcy's law where permeability is defined as a material property of the rock. This indicates that Darcy's law dramatically fails to describe scCO_2_ transport in nanochannel. Form the analysis of Fig. 2 and 3 mentioned above, we conclude that the permeability increases and the viscosity decreases as pressure gradient increases. Hence, the ratio of the permeability to the viscosity, *i.e.*, mobility, increases with the increasing of pressure gradient, characterizing the seepage capacity of scCO_2_ in nanochannel. It is observed that the mobility is linearly related to pressure gradient shown by the inset in Fig. 5. This corresponds to the hydrodynamic model of gas flow in nanoporous media where permeability is power function of pressure gradient.^33^

### 3.2 Effect of nanochannel width

#### 3.2.1 Effect of nanochannel width on adsorption of scCO_2_

**Fig. 6:**
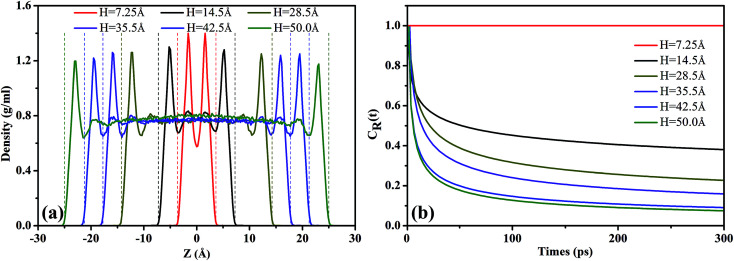
Adsorption of scCO_2_ (a) density profiles of scCO_2_ in different width of nanochannel ranging from 7.25 Å to 50.00 Å with acceleration of 0.01 kcal (Å^−1^ g^−1^). (b) Residence autocorrelation function for carbon atoms in the adsorbed layers as a function of time.

The density profiles of scCO_2_ in different nanochannels were presented in Fig. 6(a), showing the formation of several structuring layers for scCO_2_ molecules, which is consistent with previous reports.^18,24,37,60^ For *H* = 7.25 Å, the two symmetrical density peaks indicate that scCO_2_ form two primary adsorption layers due to partial superimposition of the attractive potentials from two surfaces.^37^ Accordingly, the peaks of the density profiles are the highest. As *H* = 14.5 Å, the appearing of two primary peaks and two secondary peaks indicates that there are four adsorption layers form within the nanochannel. This arises from the further separation of the attractive potentials from two surfaces. Accordingly, the primary peaks of the density profiles decrease. For *H* = 28.5 Å, bulk scCO_2_ occurs in the central area of the nanochannel apart from the four adsorption layers. The density peaks further decrease because the attractive potentials from two surfaces have been effectively separated into two single potential system. A similar phenomenon can be observed as *H* further increases from 35.5 Å to 50 Å. The results show that nanochannel size has a significant influence on the adsorption mechanism of scCO_2_ especially in very narrow channels. For narrower nanochannels, the superposition of attractive potentials from two surfaces results in strong adsorption of scCO_2_ on the surfaces. This can reduce the permeability of scCO_2_ in shale porous medium, which contributes to CO_2_ storage rather than its flow in shale reservoirs.

Fig. 6(b) shows the residence autocorrelation functions, *C*_R_(*t*), as a function of the residence time of scCO_2_ molecules remaining near the surface. As *H* increases, *C*_R_(*t*) decays fast, implying the shorter time that scCO_2_ remains in the absorbed layer. As *H* increases, the overlapping attractive potentials from two surfaces gradually separate from each other and finally form two single potential systems near the surfaces. This weakens the interaction between scCO_2_ and the surfaces resulting in the shorter residence time of scCO_2_ on the surface, and also implies a more frequent exchange of scCO_2_ appeared between the adsorption layer and bulk phase in the center of nanochannel inside the wider nanochannel. For *H* = 7.25 Å, *C*_R_(t) always equals to one indicating that all scCO_2_ molecules reside in the adsorption layer. As *H* further increases from 14.5 Å to 50.0 Å, *C*_R_(*t*) plateaus appears after about 120 ps indicating that a part of scCO_2_ molecules always reside near the surface within the simulation time. Value of *C*_R_(*t*) plateaus decrease as *H* increases, also showing the stronger adsorption for narrower nanochannels. As *H* ranges from 35.5 Å to 50.0 Å, *C*_R_(*t*) curves are basically coincident, suggesting that scCO_2_ adsorption is independent of *H* larger than 35.5 Å. This is mainly due to that attractive potentials from two surfaces have been effectively divided into two single potential systems near the channel surface. The results also demonstrate that the narrower nanochannel storages CO_2_ more securely while the wider nanochannel favors scCO_2_ transport.

#### 3.2.2 Effect of nanochannel width on diffusion coefficient of scCO_2_

**Fig. 7:**
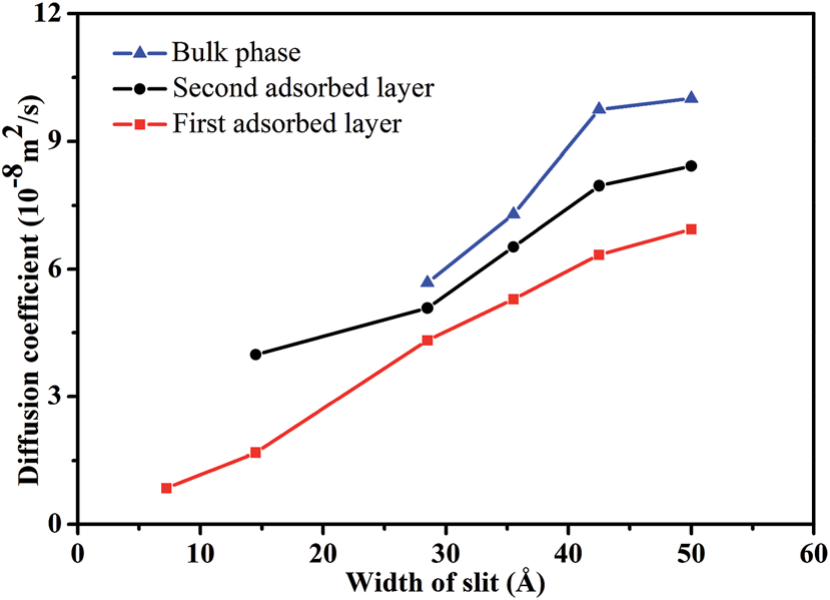
The diffusion coefficient of scCO_2_ molecular in different adsorbed layers behind various width of nanochannel.

**Fig. 8:**
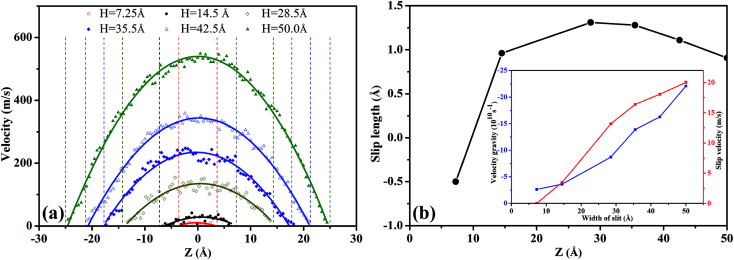
(a) Effect of nanochannel width on the velocity profiles of scCO_2_. (b) Slip length as a function of the nanochannel width for scCO_2_ molecular.

Fig. 7 demonstrated the dependence of the diffusion coefficient on *H*. The diffusion coefficient increase monotonically with *H*, indicating that scCO_2_ molecules migrate more easily in wider channels. Compared to the less constraint of bulk phase with a larger diffusion coefficient, the adsorption phase experience a stronger constraint from the surfaces which leads to a smaller diffusion coefficient. The order of diffusibility for scCO_2_ at different areas in the nanochannel is bulk phase > second adsorption layer > first adsorption layer, as described in Fig. 7. The diffusion coefficient of scCO_2_ molecules in the first adsorption layer is nonzero, which may indicate that the surface diffusion occurs by hopping of scCO_2_ molecules from one site to another.^61^ Therefore, surface diffusion is one factor that scCO_2_ molecules move more quickly in the nanochannel. This mainly results from two aspects. One is the decrease of scCO_2_ adsorption density (Fig. 6(a)), leading to the viscosity reduction. The other is the increase of scCO_2_ velocity gradient (the inset in Fig. 8(b)), indicating the enhancement of shear effect and then results in the shear thinning, which reduces scCO_2_ viscosity.

#### 3.2.3 Effect of nanochannel width on flow behavior of scCO_2_

To understand the effect of nanochannel width on scCO_2_ flow behavior, the velocity profiles and the slip lengths were plotted in Fig. 8(a) and (b), respectively. The related fitting parameters describing the flow process were provided in the ESI Table S5.†

In Fig. 8(a), the scCO_2_ velocity profiles exhibit a parabolic shape and are symmetrical about the central axis of the nanochannel. Moreover, as *H* increases, the velocity curve becomes sharper and the axial velocity of scCO_2_ increased. This is caused by the intensive constraint on the scCO_2_ molecules near the surface in the smaller nanochannel, as shown in Fig. 6. However, the bulk scCO_2_ in the central area of the wider nanochannel are rare constrained.

In Fig. 8(b), the slip length exhibits non-monotonous increase with a maximum value as *H* increases. For 7.25 Å < *H* < 14.5 Å, the slip velocity increases fast while the velocity gradient increment is small as *H* expands, as indicated by the inset in Fig. 8(b). Then the ratio of the slip velocity to the velocity gradient, *i.e.*, slip length, increases rapidly. For 14.5 Å < *H* < 28.5 Å, both the slip velocity and the velocity gradient increase rapidly. Ratio of slip velocity to velocity gradient enhances gradually and reaches the maximum value as *H* = 28.5 Å. Therefore, the slip length increases slowly and its maximum value occurs. For 35.5 Å < *H* < 50.0 Å, the increase of the slip velocity becomes slower while the velocity gradient increase rapidly. The ratio of them weakens gradually. Consequently, the slip length decreases slowly.

Fig. 8(b) also shows a negative slip length^54–57^ occurring at *H* less than about 10.0 Å. In this case, the overlapping between attractive potentials from the surfaces is so strong that a part of scCO_2_ molecules stick on the surfaces. The external pressure gradient cannot completely overcome the attractive interactions between scCO_2_ and the surface. This leads to the zero velocity near the surfaces, indicated the presence of scCO_2_ in boundary layer.

**Fig. 9:**
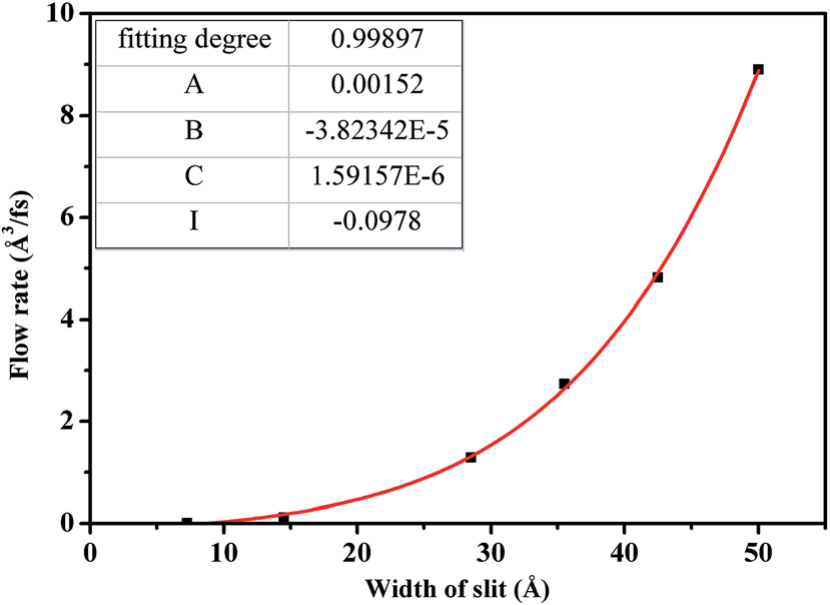
The dependence of flow rate on channel width for scCO_2_ molecular.

Fig. 9 describes the dependence of flow rate on *H*. As *H* is less than 30.0 Å, flow rate increases slowly due to the strong constraint on scCO_2_ molecules from the superimposition of attractive potentials of two surfaces. The attractive potential from the surface has a significant effect on scCO_2_ transport through nanochannel. As *H* is larger than 30.0 Å, flow rate increases rapidly because of the less constraint on scCO_2_ molecules where attractive potentials from two surfaces separate completely. Here, the attractive potential from the surface has little effect on the transport of scCO_2_ in nanochannel. Therefore, nanochannels with *H* less than 30.0 Å contribute to CO_2_ storage in shale. To quantitatively describe the dependence of the flow rate on *H*, the relationship between them was obtained by fitting data applying the power function polynomial

5 *Q* = *AH*^2^ + *BH*^3^ + *CH*^4^ + *I*

The fitting parameters were shown by the inset in Fig. 9. Compared to the continuum flow with slip effect,^52^ the fourth power of *H* appears in the fitted formula. This could be mainly due to the fact that both the viscosity and the slip length of scCO_2_ in nanochannel relate to *H*, as indicated in Fig. 7 and 8(b).

## 4. Conclusions

NEMD simulations were employed to study the scCO_2_ transport behavior within the silica nanochannel in shale. The influences of flow driving pressure gradient and nanochannel size were systematically investigated. These effects were understood by exploring density distribution of scCO_2_ molecules, residence time of scCO_2_ in the first adsorption layer, the diffusion coefficient, the velocity profile, the slippage effect, and the flow rate.

Our simulations reveal that the structuring adsorption layers of scCO_2_ molecules occur within the silica nanochannel yet under the driving of pressure gradient. As either pressure gradient or nanochannel width increases, the peaks of scCO_2_ density profiles decrease and the density of the bulk phase increases. The higher pressure gradients result in the larger shear force, which leads to desorbing scCO_2_ molecules near the nanochannel surfaces. The wider nanochannel leads to the weaker overlapping of the attractive potentials from the two surfaces, attenuating the adsorption of scCO_2_ molecules near the surfaces. The higher pressure gradients and the wider nanochannel can promote scCO_2_ diffusibility for both bulk region and the adsorption layers near the surfaces. The improvement of scCO_2_ diffusion ability may be mainly determined by the decrease of partial pressure near the surfaces, the increase of velocity of the central area in the nanochannel, and the surface diffusion of scCO_2_ hopping from one site to another. The increased diffusion ability also indicates the reduction in scCO_2_ viscosity. The negative slippage occurs at lower pressure gradients or within the narrower nanochannel is mainly due to the stronger adsorption of scCO_2_ molecules near the surfaces. The slip length exhibits non-monotonic increase and existence of a maximum value with the increase in the pressure gradient or the nanochannel size. The flow rate of scCO_2_ through the nanochannel shows a nonlinear characteristic with pressure gradient or nanochannel size. It is also found that the mobility is proportional to the pressure gradient. This implies the breakdown of Darcy's law at nanoscale because of the strong adsorption effect, surface diffusion and slippage effect.

## Associated content

Additional simulation results to determine the initial state of scCO_2_ in channel, parameters of force field and a part of fitting parameters are described in the text.

## Conflicts of interest

There are no conflicts to declare.

## Supplementary Material

RA-008-C7RA11746A-s001
