# Supplementary material for: Pressure-driven supercritical CO2 transport through a silica nanochannel
Source: RSC Adv. 2018 Jan 4;8(3):1461–8. doi: 10.1039/c7ra11746a (PMC9077126; doi:10.1039/c7ra11746a)
Supplement: RA-008-C7RA11746A-s001 [file RA-008-C7RA11746A-s001.pdf]

Electronic Supplementary Material (ESI) for RSC Advances.
This journal is © The Royal Society of Chemistry 2017

Supporting Information

Pressure-driven Supercritical CO_2 Transport through a Silica Nanochannel

Bing Liu [a,*], Xiaoqi Li [a], Chao Qi [a], Tingyi Mai [a], Kaiyun Zhan [a,*], Li Zhao [a], Yue Shen [a]

[a] School of Science, China University of Petroleum, Qingdao 266580, Shandong, China

1. Potential model

This section provides details of the potential models used in the molecular dynamics simulation. The CO_2 molecule consists of three sites, one carbon (ct) and two oxygens (o'). The cristobalite was employed to construct nanochannel with the hydroxylated surface. There are four atom types in silica: atom Si (sz) and O (oz) of SiO_2, atom H (ho) and O (oh) of hydroxyl. The nanochannel was set to rigid and was fixed in all simulations. Angle was modeled by a harmonic potential of the form:

$$U = \frac{1}{2}\kappa_r(\theta - \theta_0)^2 \tag{1-1}$$

Bond was modeled by a harmonic potential of the form:

$$U = \frac{1}{2}\kappa_\theta(r - r_0)^2 \tag{1-2}$$

The intermolecular potential is the sum of a Lennard-Jones (L-J) term and a Coulomb term:

$$U_{ij} = 4\varepsilon_{ij}\left[\left(\frac{\sigma_{ij}}{r_{ij}}\right)^{12} - \left(\frac{\sigma_{ij}}{r_{ij}}\right)^{6}\right] + k\frac{q_i q_j}{r_{ij}} \quad (1\text{-}3)$$

The following Tables present the intra- and inter-molecular potential parameters used in the simulations.

Table S1: Lennard-Jones parameters, point charges and atomic masses.

Type	σ (Å)	ε (kcal/mol)	q (e)	Mass (g/mol)
o’	3.03300	0.1598518052	-0.4238	15.999400
ct	2.75700	0.0558519312	0.8476	12.011150
sz	3.30203	0.0000018405	2.1000	28.086000
oz	3.16556	0.1554000000	-1.0500	15.999400
oh	3.16556	0.1554000000	-0.9500	15.999400
ho	0.00000	0.0000000000	0.4250	1.007970

Table S2: Bonds lengths and harmonic force constants.

Bond	κ_r (kcal/(mol·Å^2))	r_0 (Å)
ct-o’	295.4111	1.1490
sz-oz	600.0000	1.6150
sz-oh	600.0000	1.6350
oh-ho	600.0000	1.0000

Table S3: Angles and harmonic force constants.

Angle	κ_θ (kcal/(mol·rad^2))	θ_0 (deg)
o’-ct-o’	295.4111	180.0000
oz-sz-oz	600.0000	109.4700
sz-oz-sz	600.0000	155.0000
oz-sz-oh	600.0000	109.4700
oh-sz-oh	600.0000	109.4700
sz-oh-ho	600.0000	109.4700

2. Adsorption of scCO_2

To understand the adsorption of CO_2 in nanochannel under 373.15k and 35MPa, the density profiles and isothermal adsorption curve were calculated with equilibrium dynamics simulation (EMD). Two procedures were carried out. Firstly, CO_2 molecules with the density matched with the given temperature and pressure were

placed in nanochannel. The pressure of bulk phase was examined and set to the final pressure of the whole system after 2ns EMD simulation. Then, the total adsorption and excess adsorption was caculated. The results were plotted in Figure S1.

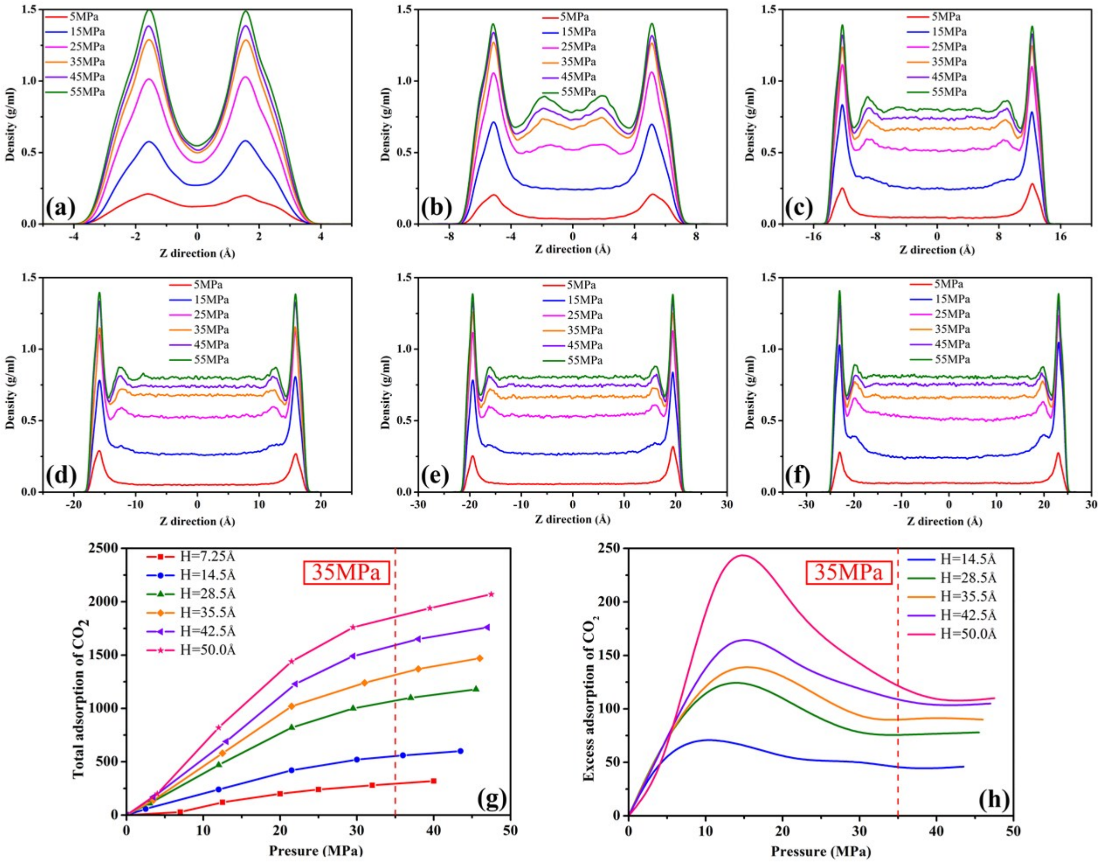

Fig S1. Density profiles of CO_2 in the nanochannel with different width under different pressure: (a) 7.25 Å, (b) 14.5Å, (c) 28.5 Å, (d) 35.5 Å, (e) 42.5 Å, and (f) 50.0 Å. The dependence of adsorption on pressure: (g) total adsorption, and (h) excess adsorption.

Figure S1a shows the density profiles of CO_2 inside the nanochannel with width of 7.25 Å under different pressures. It indicates one adsorbed layer occurring near each surfaces in the nanochannel. The increment of adsorbed molecules decayed with the increase of pressure. The similar tendency was observed in wider nanochannels. In Figure S1b, the second adsorbed layer appeared gradually with the raise of pressure in the nanochannel of 14.5 Å. The phenomenon was also observed in wider

nanochannels. Figure S1c, S1d, S1e and S1f describe the density distributions of CO_2 molecules within nanochannels larger than 14.5 Å. The results show that the second adsorbed layer appears preliminary at 15 MPa and enhances gradually with the increase of pressure.

Figure S1g and Figure S1h present the total adsorption and the excess adsorption of CO_2, respectively. It is observed that CO_2 molecules reach the saturated adsorption inside the nanochannels at 373.15 K and 35 MPa. This underlies the further study of CO_2 transport through nanochannels.

3. Fitting parameters of velocity profiles

The velocity profiles were obtained by fitting data using the quadratic function:

$$v(z) = az2 + bz + c(-14.25 \leq z \geq 14.25) \quad \text{(s1)}$$

where a, b, and c are the fitting parameters describing the flow process, a characterizes the curvature. The lager the absolute value of a is, the steeper the curve and the greater the velocity gradient. c represents the maximum velocity. The value of fitting parameters influenced by pressure gradient were shown in Table S4, and the value of fitting parameters influenced by nanochannel width were shown in Table S5.

Table S4: Values of fitting parameters influenced by pressure gradient

acceleration (kcal/(mol·Å))	*a*	*b*	*c*
0.002	-0.17459	-0.47367	32.97418
0.006	-0.41995	0.79587	96.89612
0.01	-0.72687	0.12186	156.63557
0.03	-2.55533	0.23876	568.79184
0.05	-5.40817	0.81481	1103.8837

Table S5: Values of fitting parameters influenced by nanochannel width

width of nanochannel (Å)	*a*	*b*	*c*
7.25	-0.48899	-0.85337	10.71326
14.5	-0.59683	0.72443	27.82092
28.5	-0.65869	0.64921	134.55876
35.5	-0.75796	-0.44694	234.7946
42.5	-0.78029	0.29889	344.04174
50.0	-0.88666	0.06912	539.76988
